# Emerging Role of Migration and Invasion Enhancer 1 (MIEN1) in Cancer Progression and Metastasis

**DOI:** 10.3389/fonc.2019.00868

**Published:** 2019-09-04

**Authors:** Prem Prakash Kushwaha, Sanjay Gupta, Atul Kumar Singh, Shashank Kumar

**Affiliations:** ^1^Department of Biochemistry, School of Basic and Applied Sciences, Central University of Punjab, Bathinda, India; ^2^Department of Urology, Case Western Reserve University, Cleveland, OH, United States; ^3^The Urology Institute, University Hospitals Cleveland Medical Center, Cleveland, OH, United States; ^4^Department of Nutrition, Case Western Reserve University, Cleveland, OH, United States; ^5^Divison of General Medical Sciences, Case Comprehensive Cancer Center, Cleveland, OH, United States; ^6^Department of Urology, Louis Stokes Cleveland Veterans Affairs Medical Center, Cleveland, OH, United States

**Keywords:** MIEN1, cancer, metastasis, invasion, posttranslational modification

## Abstract

Tumor metastasis is a sequential event accounting for numerous cancer-related fatalities worldwide. The process of metastasis serially involves invasion, intravasation, extravasation, and tumor growth at the secondary site. Migration and invasion enhancer 1 (MIEN1) is a membrane associated protein overexpressed in various human cancers. Biological activity of MIEN1 is driven by geranylgeranyltransferase-I mediated prenylation at CAAX motif and methylation of the prenylated protein that anchors MIEN1 into the cellular membrane. Post-translationally modified MIEN1 interacts with Syk kinase and Annexin A2 protein; polymerizes G-actin and stabilizes F-actin filament; induces focal adhesion kinase phosphorylation and decrease cofilin phosphorylation implicated in both invasion and metastasis of different cancer types. In the present review, we discuss the structure, function, and involvement of MIEN1 in cancer progression. We also highlight the future prospects of MIEN1 as an emerging molecule and novel target in cancer cell invasion and metastasis.

## Introduction

Metastasis is an intricate process of cell dissemination that primarily depends on the ability of tumor cells to detach from basement membrane through dynamic reorganization of the cytoskeleton proteins. MIEN1 has been identified as a primary regulator of cancer cell migration and invasion. The protein is overexpressed in human breast, prostate, colorectal, gastric, ovarian, squamous cell carcinoma and non-small cell lung cancer (NSCLC) (1–7). Emerging literature suggest MIEN1 as a new tumor-specific target protein as it facilitates cancer progression that plays key role in distinct processes of migration/invasion of cancer cells. MIEN1 protein has several aliases such as hepatitis B virus (HBV) X-Transactivated Gene 4 Protein, HBV XAg-Transactivated Protein 4, C17orf37 (Chromosome 17 Open Reading Frame 37), RDX12, XTP4, ORB3, and C35. In this review, we discuss the structure, function, expression and involvement of MIEN1 in cancer progression. We also highlight future prospects of MIEN1 as novel drug target against cancer cell invasion and metastasis.

## Structure of MIEN1

**Figure 1:**
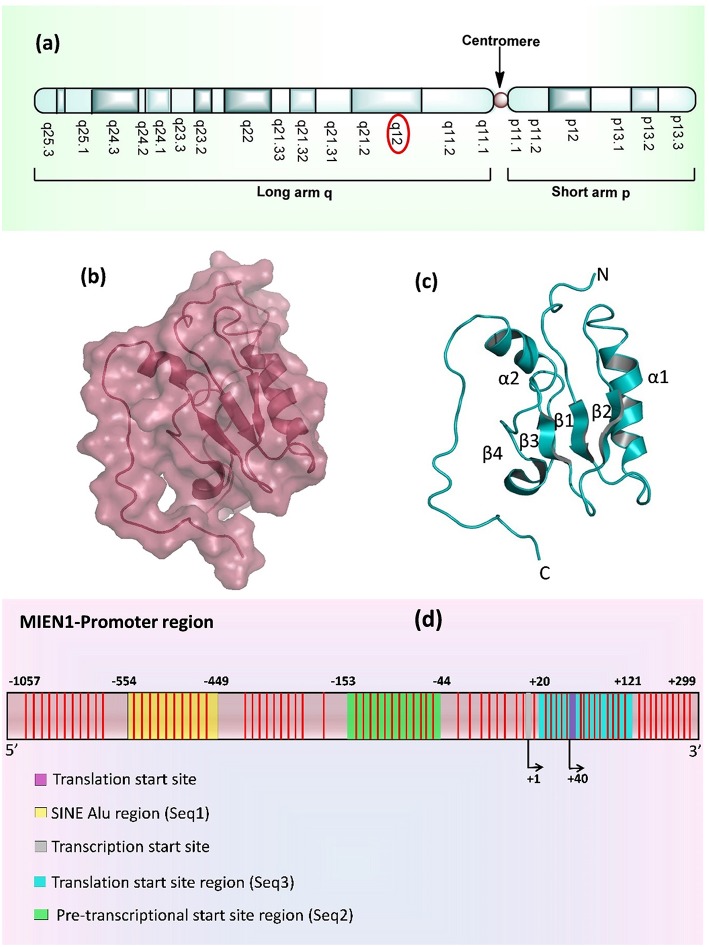
**(a)** Location of MIEN1 gene on chromosome 17 (q12 position) **(b)** Surface structure of the MIEN1 protein (PDB: 2LJK), **(c)** Ribbon representation of MIEN1 protein having α (α1 and α2) helices and β (β1, β2, β3, and β4) sheets; and N- and C-terminus **(d)** MIEN1 methylated promoter sequence. CpG islands are represented in red lines.

MIEN1 is a membrane-anchored protein located in the 17q12 region of the human chromosome which is 505 nucleotide upstream to 3′ ERBB2 oncogene, inside the hot-spot locus of cancer (Figure 1a) (1, 8). *MIEN1* encodes 115 amino acids and 12 kDa molecular weight protein. The spot contains multiple genes which are implicated in cancer initiation and development. Solution structure of MIEN1 reveals the presence of thioredoxin-like fold having a redox-active motif. Four stranded β-sheet and two α-helices forms a central α/β core domain of the protein (9). Two helices (α1 and α2) are comprised of Phe35-Gln49 and Glu86-Asn98 residues. Four β-strands (β1, β2, β3, and β4) ranges from Arg24 to Tyr29, Glu54 to Leu59, Phe65 to Glu68 and Val74 to Ser76 residues respectively (Figures 1b,c). MIEN1 hydrophobic core constitutes of Tyr39, Leu42, Val46, Tyr50, Phe65, Val74, Leu89, and Ile (53, 67, 69, 90, and 93 positions) residues. Approximately 15 residues at each N- (Met1-Glu16) and C- (Glu103-Leu115) terminal of the protein present an unordered structure. ^1^H-^15^N heteronuclear NOE experiments exhibited that MIEN1 backbone is flexible due to a lack of stabilizing long-range interactions with the rest of the protein (9). MIEN1 protein has reduced oxidative state, however, the oxidation state does not impede its protein activity. CXXC motif, present at C-terminus is directly involved in oxidation-reduction reaction. A cluster of some aromatic residues at MIEN1 active site might be responsible for overall protein structure stabilization and its binding interaction partners. The CXXC motif and aromatic amino acid cluster are located apart from each other (9). The backbone residues of MIEN1 in both oxidized and reduced state is observed to be within the same conformation, but differences in the active motif region were found due to amide resonance. MIEN1 possess a disulfide bond in its active sites. However, reduction of the active-site disulfide bond during reduction to oxidation state does not affect MIEN1 folding pattern (9). The MIEN1 oxidation-reduction potential plays critical role in AKT phosphorylation (9).

MIEN1 promoter region is comprised of several CpG islands, CpG dyads and a short interspersed nuclear element (SINE) Alu repeat. Alu are the short stretch of DNA, that form the Alu family of repetitive DNA elements. Alu elements of more than 300 base pairs are known as SINEs. Approximately, one-third of the total CpG sites (methylation sites) in the genome resides in Alu elements. Normally Alu elements are methylated and transcriptionally inactive (10, 11). Various sites in the Alu element of MIEN1 promoter region remain hypermethylated in the normal cells (12). The SINE Alu elements belong to a category of gene regulatory elements known as retrotransposons that have been shown to become less mobile upon genetic regulation (13–16). Genome wide study has established that SINEs are present in most human cancers near to transcriptional start sites (TSS), which are hypomethylated in cancer (15, 17). SINEs enriched regions present at about 550 bases from the TSS of MIEN1. The methylation landscape and promoter region of MIEN1 is shown in Figure 1d.

## MIEN1 Expression

**Figure 2:**
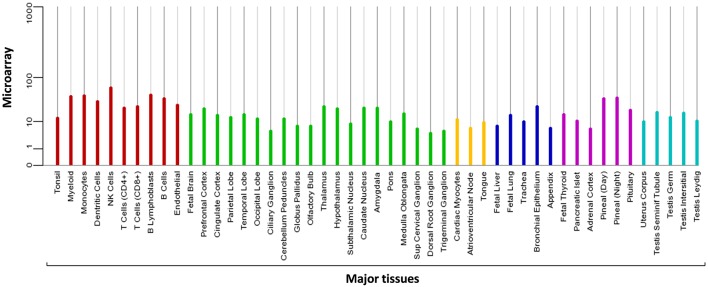
Microarray data representing the expression pattern of MIEN1 in various tissues. The measurements were obtained for 76 normal human tissues and compartments hybridized against HG-U133A. The Affymetrix MAS5 algorithm was used for array processing and probe sets were averaged per gene [https://www.genecards.org/cgi-bin/carddisp.pl?gene=MIEN1].

MIEN1 is predominantly present in the cytosol with little plasma membrane-associated expression. The biological function of MIEN is to increase cell migration by inducing filopodia formation at the leading edge of migrating cells (18). It also plays a key role in regulation of apoptosis, possibly through control of CASP3, and is involved in redox-related process. Microarray analysis demonstrates the expression of MIEN1 in various compartments in the blood, brain, liver, pancreas, lung, thyroid, and testis (Figure 2). Overexpression of MIEN1 was initially identified in breast and colon cancer. MIEN1 expression positively correlates with the grade and stage of breast cancer compared with minimal expression in normal tissues (1). Thus, the differerential expression of MIEN1 in normal vs. cancer cells proposes it to be a novel tumor biomarker. In patients with metastatic breast cancer, aberrant expression of MIEN1 has been noted in distant metastatic sites such as lungs and liver, suggesting a possible role of MIEN1 protein in metastatic dissemination of tumor cells (1, 7). In prostate cancer, MIEN1 is overexpressed in the higher grades of prostate adenocarcinoma compared with low expression in normal or benign prostatic tissue (2). Studies indicate that MIEN1 overexpression is linked to genomic amplification of the ERBB2 locus, since its sufficient expression is noted in the ERBB2 nonamplified breast and prostate tumors, suggesting that MIEN1 might have an independent functional promoter (19).

## Posttranslational Modification of MIEN1

Modification of eukaryotic cellular protein with isoprenoid unit is known as prenylation. Protein prenylation involves transfer of either a farnesyl or a geranyl-geranyl moiety to C-terminal cysteine(s) of the target protein. This process mediates protein–protein interactions and protein–membrane interactions having decisive function in a biological system. Three different enzymes are known to be involved in protein prenylation, namely Ras converting CAAX endopeptidase 1 (RCE1), geranylgeranyltransferase (GGTase 1) and farnesyltransferase (FTase). The cysteine residue of CAAX motif is modified by either farnesylation (15 carbon chain addition by protein farnesyltransferase enzyme/FTase) or geranylgeranylation (addition of 20-carbon chain by GGTase-I) during prenylation (20, 21). Most of the prenylated proteins belong to the CAAX family, which are later post-translationally altered by the addition of isoprenyl groups. CAAX denotes a protein comprised of a CAAX motif at the C-terminal; where “C” is the cysteine amino acid that acts as the isoprenoid accessory site; “A” denotes one of the amino acid from Ile, Val, Leu, Ala, Met and Pro; and “X” signifies any of 22 amino acids (22). Farnesyltransferase (EC 2.5.1.58) is the prenyl transferase group enzyme, which adds fifteen-carbon isoprenoid (farnesyl group) to CAAX motif of the proteins. Geranylgeranyltransferase (GGTase 1) is another prenyl transferase group which adds twenty-carbon isoprenoid (geranylgeranyl group) to the CAAX motif of proteins (23–25). CAAX protein sequence, together with prenylated motif, determines the different localization of the protein, at plasma membrane/endomembrane or at a cytosolic location (26). Therefore, any modification at the C-terminus of these proteins might affect their stability, function, localization, and protein-protein interaction. The regulatory capacity of prenylated proteins plays confirmatory role in cellular signaling and pathophysiological conditions. The prenylation of several oncogenes including RAS GTPases, is known to be involved in cancer progression through migration, invasion, increased proliferation and filopodia formation (27).

MIEN1 has a functional isoprenylation “CAAX” motif at the C-terminal tail that is post-translationally modified by geranyl-geranyl transferase-I (GGTase-I), a cytosolic enzyme at Cys-112 (18). Geranylgeranylation of MIEN1 at the CAAX motif supports its anchorage to the cytosolic part of the plasma membrane. A study demonstrated that MIEN1 prenylation is accountable for the filopodia formation and potential directional migration (18). An interesting feature of membrane anchored MIEN1 is the existence of a consensus sequence for prenylation comprising of the last four amino acids, CVIL, at the C-terminal end (Table 1).

**Table 1:** Thirty amino acid sequence of MIEN1 and other illustrative geranylgeranylated proteins.

**Geranylgeranylated protein**	**Carboxyl terminal sequence**
Rho A	KDGVREVFEMATRAALQARRGKKKSG**CLVL**
Rho B	VREVFETATRAALQKRYGSQNGCINC**CKVL**
Rho C	KEGVREVFEMATRAGLQVRKNKRRRG**CPIL**
Rac 1	RGLKTVFDEAIRAVLCPPPVKKRKRK**CLLL**
Cdc42	QKGLKNVFDEAILAALEPPEPKKSRR**CVLL**
MIEN1	EKDLIEAIRRASNGETLEKITNSRPP**CVIL**

*The CAAX motif is in bold and underlined*.

**Figure 3:**
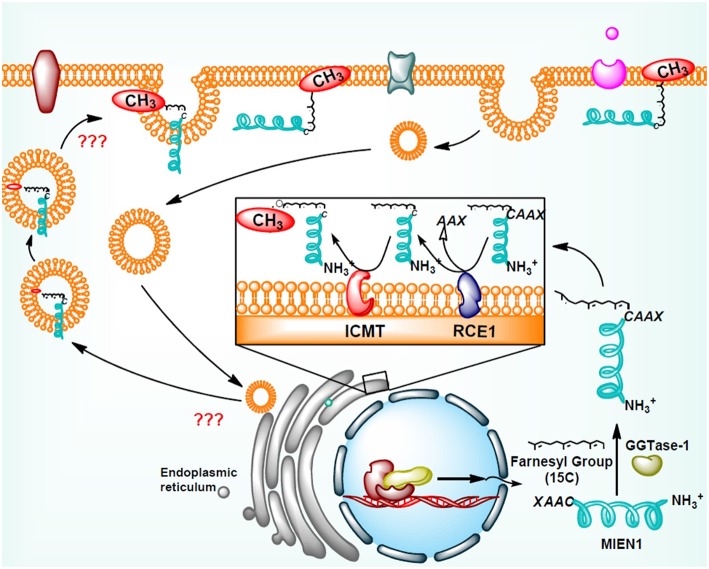
Post-translational modification of MIEN1 protein by protein geranylgeranyltransferase-I (GGTase-I).

Addition of isoprenyl group in the MEIN1 protein leads to a two-step supplementary post-prenylation process including cleavage of the last three amino acid at C-terminus by an endoprotease enzyme, Rce1 (Ras-converting CAAX Endopeptidase 1) and prenylated-cysteine methylation executed by ICMT (isoprenylcysteine-O-carboxyl methyltransferase) enzyme (18) (Figure 3). Galectins are another family of proteins that bind to activated RAS proteins by interacting the farnesyl lipid and an adjoining RAS region thereby stabilizing their membrane anchorage and enhance signaling (28). Likewise the area is open to identification of protein(s) which may stabilize the membrane-MIEN1 association.

## Methylation and Demethylation of MIEN1

Dasgupta et al. demonstrate for the first time that MIEN1 protein undergoes prenylation during post translational modification (18). To-date, there is no information available regarding the methylation and demethylation of prenylated MIEN1 protein. It is known that MIEN1 protein, like RHO family and Cdc-42 proteins, have CAXX motif at the C-terminus. RCE1 enzyme removes AXX from the CAXX motif of these proteins, whereas the left C-terminus is methylated having a higher potential to attach to the cellular membrane. Reports suggest that MIEN1 is a membrane associated protein and its biological activity is dependent upon prenylation and subsequent methylation at the C-terminus of the CAXX motif (18). Post-translational modification of prenylated proteins having a CAAX motif changes the hydrophilic nature of the protein toward lipophilicity, which facilitates their attachment to plasma membrane (18). The study further reported that prenylation of MIEN1 protein is associated with filopodia formation and directional migration. Thus, the regulatory mechanism involved in the methylation/de-methylation of the protein might suggest it to be a potential target for different cancers.

## MIEN1 and Metastasis

**Figure 4:**
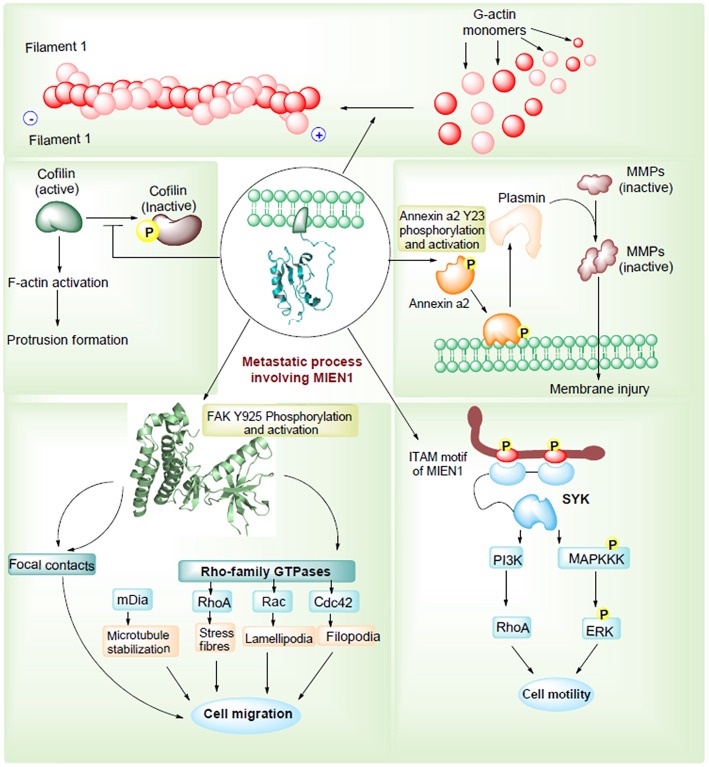
MIEN1 enhances migration and invasion via different routes.

Epithelial-mesenchymal transition (EMT) permits tumor cells to dedifferentiate enhancing the invasive potential during the metastatic process. During EMT, expression of adhesion molecules, such as integrins and cadherins, and their receptors are involved in increased cellular motility in tumor cells (29). Besides, metalloproteases, cytokines and chemokines are important factors that regulate cell motility and metastasis. Annexin A2 (AnxA2) is a Ca^2+^ dependent phospholipid binding protein that acts as an extracellular proteolytic molecule regulating plasmin generation. Plasmin modifies the tumor microenvironment by enhancing the extracellular matrix protein degradation and release of growth factors, which are all associated with cancer progression (30). Higher levels of AnxA2, in association with STAT3, regulates proliferation, invasion, and migration of cancer cells. MIEN1 overexpression increases AnxA2 phosphorylation at Tyr23 residue, which stimulates cell surface translocation and catalyzes activation of proteolytic activity of AnxA2 (31). Thus, MIEN1-AnxA2 protein-protein interaction might be regarded as one of the mechanisms facilitating tumor metastasis (Figure 4). Katz et al. demonstrated that Syk kinase, a downstream mediator of MIEN1 signaling mediates breast epithelial cell transformation in 3D cultures (32). In the same study, high expression of MIEN protein was associated with various transformation events, such as colony formation, invasion and large acinar formation. Besides transformation, EMT changes were also observed including a decreased expression of E-cadherin and keratin-8 (33). Actin cytoskeleton has been extensively known for its importance in metastasis. Recently, MIEN1 protein was found to localize in a concerted manner beneath the action protrusion structures of migratory cancer cells (Figure 4). The study reported that MIEN1 levels regulate the actin-protrusive structures in migrating cells (34). MIEN1 is also involved in the F-actin polymerization (from G-actin monomers) and stabilization. MIEN1-mediated increase in focal adhesion kinase (FAK) and decrease in cofilin phosphorylation at Tyr-925 and Ser-3 are associated with actin dynamics and cellular adhesion in migratory cancer cells (34).

**Figure 5:**
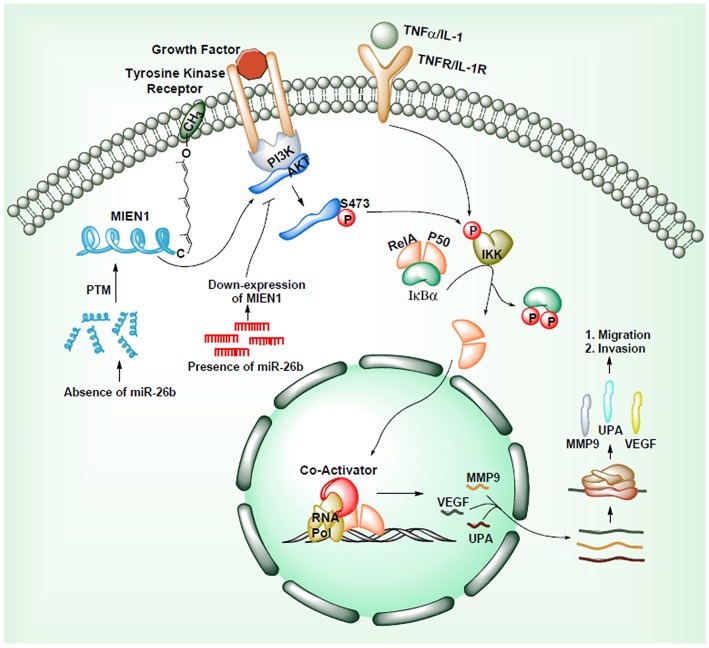
MIEN1 enhances Akt phosphorylation and NFκB signaling.

Another mechanism through which MIEN1 regulates invasion and metastasis is via the PI3K/Akt pathway (35). Activation of Akt can lead to NF-kB activation resulting in an increase in matrix metallopeptidase 9 (MMP-9), urokinase plasminogen activator (uPA) and vascular endothelial growth factor (VEGF). These are key proteases and angiogenic factors that are downstream of NF-kB pathway, facilitating migration and invasion of cancer cells. Studies have shown that the absence of miR-26b results to post translational modification of the MIEN1 to generate mature/functional protein. Overexpression of MIEN1 induces the PI3K/Akt pathway via phosphorylation of the S473 residue of Akt proteins. Ultimately phosphorylation of IκBα protein allows the release of RelA and P50 proteins, which can then translocate into the nucleus, interact with different coactivators and enhance the expression of invasion and migration gene. In the presence of miR-26b, decreased levels of MIEN1 functional protein results in reduced migration and invasion (Figure 5). Overall, in most studied cancers (1–7), MIEN1 plays a critical role in tumor dissemination and represents a potential target of metastasis in cancer cells.

## Association of MIEN1 With Other Proteins

**Figure 6:**
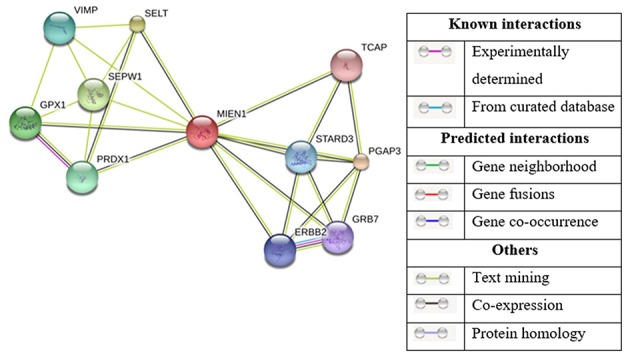
Based on the STRING database, the network of MIEN1 and other protein interactions. Ten differentially expressed proteins formed a network map with the MIEN1 protein.

The STRING Database has been used to establish possible interactions between other proteins. In this analysis, 10 proteins were recognized by interaction analysis and a network mapping with MIEN1 (Figure 6). The possible interaction or association of MIEN1 is shown in Table 2. String database demonstrates direct interaction between MIEN1 and other proteins, such as TCAP, STARD3, PGAP3, GRB7, ERBB2, PRDX1, SEPW1, GPX1, VIMP, and SELT, respectively. It should be noted that these interactions are based on co-expression and text mining predictions (Figure 6). Further studies should be designed to evaluate the interactive response of MIEN1 with other proteins.

**Table 2:** Possible interaction or association of MIEN1 with other proteins and possible outcomes.

**Protein**	**Function**	**Association with MIEN1 protein**	**Mode of action**	**References**
PGAP3	Glycosylphosphatidylinositol (GPI) anchors are used for the attachment to cell membrane by different proteins. They are synthesized in endoplasmic reticulum and associated with particular protein in the Golgi complex. Mature GPI contains diacylglycerol (DAG) with a long-chain fatty acid/ceramides at sn-2 position (unsaturated fatty acid in newly synthesized GPI). This GPI remodeling is necessary for GPI-anchored proteins to get them attached with cellular membranes. PGAP3 probably required for PLA2 activity that eliminates an acyl-chain at the sn-2 position of GPI-anchors during GPI remodeling.	Inverse relation between PGAP3 and MIEN 1 proteins in Inflammatory bowel disease (IBD).	NS	(19, 36)
SELT	SELT have thioredoxin reductase-like oxidoreductase activity and involved in neuro biology (involved in stress mitigation, neuroendocrine secretion and Ca^2+^ mobilization). Beside that it play role in glucose homeostasis, insulin secretion, and muscle contraction etc.	SelT selenoprotein domain from *Pseudomonas fluorescens* was studied for sequence similarity with MIEN 1. About 24% sequence similarity with Z-score >7 has been reported.	NS	(9, 37)
SEP1	SEP1 is a glutathione dependent antioxidant. It is involved in redox chemistry related biological process and have role in selenium deficiency related myopathy.	MIEN1 have motif homologous to active site of selenoprotein W, but it lacks selenocysteine amino acid.	NS	(1, 9, 38)
GPX1	GPX1 defends the hemoglobin in erythrocytes from oxidative damage of peroxiredoxin 1 and also involved in redox regulation of the cell.	Interaction reported in STRING database but study was not found in scientific databases.	NS	(39)
VIMP	VIMP includes deprivation of misfolded (ER) luminal proteins	Interaction reported in STRING database but study was not found in scientific databases.	NS	(40)
STARD3	It is also known as metastatic lymph node 64 protein (MLN64). STARD3 transports cholesterol and promotes steroidogenesis in both brain and placenta.	MIEN 1 gene expression (with some other genes ERBB2, GRB7, STARD3) was reported in about one fourth studied gastric cancer cases. The study dealt with the amplification of 17q12 region, frequently involved in gastric cancer.In another study (designed for the same locus i.e., 17q12) over expression of MIEN 1 and STARD3 was found in HER2-positive breast tumors. Study demonstrate the coregulation of MIEN 1 and STARD3 by HER2. Complex of sense-antisense gene pair (CSAGP) are involved in cancers and other diseases/disorders. The genes involved in this complex shares loci with their two or more antisense partners. A study was designed to assess the reproducible co-regulatory transcription pattern of some genes with ERBB2 amplicon core genes (which included *ERBB2, GRB2, STARD3 etc*.) in breast cancer tumor samples. Gene expression of both MIEN 1 and *STARD3* was noteworthy correlated with almost all the study CSAGP genes.	NS	(41–43)
TCAP	Muscle assembly regulating factor TCAP which facilitates the antiparallel assembly of titin molecules	High expression of TCAP and MIEN1 proteins positively correlated with breast cancer. Study was based upon the amplification of a breast cancer susceptible locus (A 400-kb *ERBB2* amplicon). TCAP and MIEN 1 promoter are found nearby in the locus.	NS	(44)
ERBB2	The gene encodes EGF receptor family proteins. This protein (no ligand binding domain present) bind strongly to other ligand-bound EGF receptors forms a heterodimer, stabilize the ligand binding and enhance kinase-mediated signaling pathways.	Interaction reported in STRING database but study was not found in scientific databases.	NS	(45)
GRB7	GRB7, an adapter protein interacts with receptor kinases cytoplasmic domain which results into modulation of down-stream signaling (STAT3, AKT1, MAPK1 and MAPK3 proteins) and cell proliferation/migration.	MIEN 1 gene is situated on 17q12 position on chromosome bounded by both *Grb7* and *ERBB2* genes. Moderately decreased (about 2-fold) expression of *MIEN1 and GRB7* was found in biopsies from inflamed colonic IBD mucosa compared with noninflamed samples.	NS	(18, 19)

*PGAP3, Post-GPI attachment to proteins 3; GRB7, Growth factor receptor-bound protein 7; ERBB2, V-erb-b2 erythroblastic leukemia viral oncogene homolog 2; TCAP, Titin-cap; STARD3, StAR-related lipid transfer (START) domain containing 3; VIMP, VCP-interacting membrane protein; SELT, Selenoprotein T precursor; GPX1, Glutathione peroxidase 1; SEP1, Selenoprotein W1; NS, Non-studied; PLA2, Phospholipase A2*.

## MIEN1 and Drug Resistance

Little information is available regarding MIEN1 and drug resistance. Upregulated Dnp73, an isoform of p53 is associated with increased tumor potential. Leung et al. reported that MIEN1 in association with Dnp73 promotes tumor development and its involvement in cisplatin resistance in cancer cells (5).

## MIEN1 and Cancer

MIEN1 is overexpressed in several human cancers including breast, prostate, colorectal, gastric, ovarian, squamous cell carcinoma and non-small cell lung cancer (NSCLC), therefore playing an important role in biological processes underlying tumor metastasis. Epigenetic and genetic studies performed on MIEN1 in various human cancers have been summarized in Table 3. Loss or gain of MIEN1 activity, and its relationship with metastasis and invasions in different cancer cells and tissue specimens is shown in Tables 4, 5, respectively. Below we summarize studies which shed light on the involvement of MIEN1 protein in various cancers.

**Table 3:** Expression of MIEN 1 in different cancer and its output.

**Cancer type**	**MIEN1 up/down**	**Method of Detection**	**Source of regulation**	**Output**	**References**
Prostate	↓	*In silico* algorithms and microarray	hsa-miR-940	miR-940 mediated regulation of MIEN 1 resulted into decreased migration and invasiveness of cancer cells.Anchorage-independent growth ability was attenuated.E-cadherin overexpression and repressed EMT transition	(46)
		Bisulfite pyrosequencing	MIEN1 promoter methylation inhibition by nucleoside analogs and non-nucleoside inhibitors	Sequencing demonstrated that MIEN1 promoter have SINE Alu repeat.Study indicated that in cancer cells, due to hypomethylation of SINE Alu provides USF binding which results in MIEN1 expression.	(12)
		*In silico* analysis and ChIP	Inhibition DNA methyltransferases using RNAi.	Identified a sequence upstream of the TSS of MIEN 1 promoter that binds with USF factors.	(12)
NSCL	↓		miR-26b	miR-26b reduces MIEN1 mediated NSCLC metastasis via NF-kB/MMP-9/VEGF pathways.	(7)

*TSS, Transcription start site; RNAi, RNA interference*.

**Table 4:** Effect of loss/gain of MIEN1 protein and its effects on metastasis and invasiveness in different cancer.

**Cancer type**	**Loss/gain of function experiment**	**Invasion and migration**	**Other output**	**References**
Prostate	siRNA mediated down regulation	↓	Inhibition of transcription factor NF-κB binding with DNA reduces metastatic related genes such as MMP-9, uPA, and VEGF.!!! Reduced phosphorylation of PKB/Akt suggested that MIEN 1 increases invasive potential of prostate cancer by NF-κB mediated downstream target genes.	(2)
Breast	siRNA mediated down regulation	–	Apoptosis was detected in T47D.	(47)
	siRNA mediated knockdown	↓	Loss of actin-protrusive assemblies and decreased the cell-substratum adhesion.	(34)
	shRNA mediated down expression	–	Showed better prognosis in invasive breast cancer patients.	(48)
Oral	MIEN1 knockdown	↓	Decreased filopodia formation was observed in OSC-2 cells.	(6)
	MIEN1 overexpression	↑	Increased filopodia formation and up-regulated Akt/NF-kB effectors was reported in DOK cells.	(6)
			Deep immunohistochemical staining was found in diseased samples in evaluation to ordinary samples. Study concluded that MIEN 1 expression is positively correlated with grade cancer stage and smoking.	(6)
NSCL	siRNA mediated knockdown	↓	miR-26b reduces MIEN1 mediated NSCLC metastasis via NF-kB/MMP-9/VEGF pathways.	(7)

**Table 5:** MIEN 1 expression pattern in various cancer cell lines and tissue samples and their research outcomes.

**Cancer**	**Cell line/tissues**	**↑/↓ MIEN1**	**mRNA technique**	**Protein technique**	**Outcome**	**References**
Breast	BT-20, BT-474, SK-BR-3, MEL1700, H16N2, HCC-70, HCC-1937, MCF-7, T47D, MCF7, MCF10A, MCF10AT, MCF10CA1a, MCF10CA1d, CF10CA1h, MDA-MB-231, MDA-MB4-36, NIH3T3, 21MT-1, 21MT-2, 21NT, 21PT	↑	NB and RT-PCR	Immunohistochemical, Western Blot	Up expression was correlated with metastasis in breast cancer. In normal breast cells, MIEN 1 expression levels was comparatively low. Induces FAK phosphorylation (Tyr-925) and reduces cofilin phosphorylation at Ser-3 residue. This results in cancer cell migration. Maintains the plasticity of the dynamic membrane-associated actin cytoskeleton in term of F-actin polymerization, stabilization and ultimately increases cell motility.	(1, 34, 47)
	Primary hBC specimens (*N* = 122)				Involved in cell transformation related to invasion and metastasis characteristics. Interaction between ITAM motif of MIEN 1 and Syk kinase mediated downstream signaling was depicted.	(32)
	Her2 and luminal B subtypes of breast tumor			FRET	MIEN1 expression negatively correlates with disease free survival. MIEN1 and AnxA2 contact mediated tumor cell motility was observed.	(31)
	hBC (*N* = 40) and ANTS (*N* = 10)			Immunohistochemical, Western Blot	Upregulated MIEN1 suppressed the expression of MMP9 by PKB/Akt expression downregulation. About 80% increase in MIEN 1 expression in evaluation to ordinary ANTS. MIEN 1 level was completely interrelated with age, tumor grade, metastasis of the patient and negatively correlated with overall survival of the patient.	(48)
	Human breast cancer (*N* = 68) and Normal breast tissue samples (*N* = 20)	↑			About 83% of the breast cancer samples showed increased MIEN 1 expression. MIEN 1 expression was completely correlated with tumor node metastasis and grading. No correlation observed between MIEN 1 expression with patients age, menstrual status and tumor diameter. MIEN 1 expression was completely correlated with HER-2 receptor expression, whereas negatively correlated with ER and PR expression.	(49)
Prostate	DU-145, LNCaP C4-2, RF and UR prostate cancer cells, LNCaP, PC-3, DU-145, LNCaP-R, and PC-3 DU-145, PC-3, and LNCaP	↑	Quantitative reverse transcription-PCR, northern blotting, *in situ* hybridization	Western immunoblot, IHC, ELISAConfocal and TIRF microscopyimmunofluorescence	Expression level was associated with grade and cancer stage progression, enhanced migration and invasion. Cellular localization showed predominant expression in cytosol with deep staining in the cell membrane. miR-940 inhibited MIEN 1 mRNA degradation demonstrated decreased migration, invasiveness, attenuation in anchorage-independent growth and increased E-cadherin expression.	(2, 12, 46)
Colorectal	CC tissue sample (*N* = 65), CC tissue sample (*N* = 55) and CC tissue sample (*N* = 10)	↑	Reverse transcription-PCR (RT-PCR) and real-time PCR	IHC	Increased MIEN 1 expression was detected in CRC tissue sample in evaluation to ordinary ANTS. No expression of MIEN 1 was detected in normal colorectal tissue samples. MIEN 1 expression level was completely interrelated with tumor invasion, lymphnode metastasis and advance stage of the disease.	(3)
NSCL (Non-small cell lung)	Human lung cancer (*N* = 30) and Lung cancer ANTS (*N* = 30) A549, 95D, H520, HBE cell line	↑	Quantitative RT-PCR	Western blot	miR-26b reduces MIEN1 mediated NSCLC metastasis via NF-kB/MMP-9/VEGF pathways	(7)
Colon	Lovo and HT29	↑	qRT-PCR	Western blotting	Simvastatin (HMGCoA inhibitor) dependent down regulation of MIEN 1 at both mRNA and protein level	(50)
Oral	HOK-16B, DOK, SCC-25, OSC-2	↑			MIEN 1 up expression was allied with increased invasion and migration	(6)

*NB, Nothern blot; RT-PCR, Real time polymerase chain reaction; FRET, Forster resonance energy transfer; CC, Colorectal sample; hBC, Human Breast Cancer; ANTS, Adjacent normal tissue sample*.

### Breast Cancer

Evans et al. used immunohistochemical staining technique to study C35 expression in human breast cancer and normal tissue specimens (1). The study revealed the excessive expression levels of the protein throughout, starting from tumor initiation indicated by DCIS (ductal carcinoma *in situ*) and LCIS (lobular carcinoma *in situ*) to late stage metastasis. Approximately 50% of the studied samples exhibited high MIEN1 levels especially in the distant metastasis specimens. These specimens also showed poor prognosis characterized by lymphocytic infiltration and ERBB2 positivity. The study indicates that MIEN1 might act as a potential early detection biomarker in breast cancer. In addition, MIEN1 levels were detected in distant tissues, mainly bone, brain, liver and lung which strengthen the role of MIEN1 during disease progression (1). Additional studies provide evidence for aberrant expression of MIEN1 in different stages of breast cancer with low and/or absence of expression in normal tissues, indicating its development as a potential biomarker. Previous studies describe the involvement of ITAM motif containing protein in mammary epithelial cell transformation and mammary carcinoma development (51–53). ITAM-transformed cells with the support of its downstream Syk signaling activation is closely linked to EMT progression in cancer cells (32). A recent report identified that MIEN1 protein contains ITAM motif and is involved in metastasis through interaction with Syk protein (32). Further screening of 122 invasive breast cancer specimens using microarray technique demonstrated high levels of MIEN1 protein in these tissues, having a positive correlation with HER2 expression (32). Other studies, such as association of MIEN1 with actin cytoskeletal dynamics, ITAM and AnxA2-mediated metastasis in breast cancer progression have been previously documented (31). Treuren et al. generated MIEN1 knockout (KO) breast cancer cell lines using CRISPR/Cas9 (Clustered Regularly Interspaced Short Palindromic Repeat–CRISPR associated protein 9) system. Authors reported that MIEN1 gene deletion has no effect on the proliferation and survival of breast cancer cells, necessitating further studies to understand the role of MIEN1 in breast cancer metastasis (54).

### Prostate Cancer

Deregulation of MIEN1 protein level in prostate cancer was first reported in 2009 (2). The study demonstrated high expression of MIEN1 in prostate cancer cell lines and tumors in comparison to normal tissues (low expression) (2). The study established the role of MIEN1 overexpression with migration and invasion of prostate tumor cells mediated *via* Akt/NF-kB signaling and activation of downstream effector proteins. Akt phosphorylation at Ser473 subsequently phosphorylates IκB and IκBa in a sequential manner (Figure 5). Phosphorylation of IκBa leads to its degradation releasing NF-κB homo/heterodimers to translocate into the nucleus. MIEN1 overexpression enhances metastasis by augmenting NF-κB activation and DNA binding potential resulting in increased expression of its downstream target genes (2, 35).

Non-coding RNA is a fully functional ribonucleic acid that does translate into functional protein. Non-coding RNAs are highly dysregulated in cancer and play an imperative role in disease progression (46). Rajendiran et al. demonstrated an association of miRNA and MIEN1 protein in prostate cancer (46). The study revealed that MIEN1 is an instantaneous target of miR-940 and its downstream effectors, resulting in an alteration in mesenchymal-to-epithelial transition. The study established mir-940 as a novel modulator of MIEN1 (46). In another study, Rajendiran et al. reported the DNA methylation is scattered in the SINE Alu region of the MIEN1 promoter (12). In normal cells, SINE Alu region is hypermethylated leading to subjugation of the MIEN1 gene. In cancer cells, tumor suppressor genes, including genes involved in apoptosis, cell-cycle regulation, and hormonal regulation are hypermethylated. Whereas, oncogenic genes are hypomethylated. The hypomethylation of a section of SINE Alu region opens the chromatin structure, facilitating the ligation of USF (upstream stimulatory factor) to MIEN1 promoter, thereby resulting in its expression. The role of MIEN1 expression affecting apoptosis in prostate cancer cells has not yet been determined.

### Lung Cancer

Non-small cell lung cancer (NSCLC) is the major subgroup of lung cancer, which is one of the most common cancers among men and women. MIEN1 mRNA is shown to be overexpressed in NSCLC and correlates with poor prognosis in NSCLC patients, suggesting that MIEN1 expression could be developed as a prognostic marker (7). Li et al. reported that siRNA-mediated MIEN1 knockdown in lung cancer cells reduces the events of metastasis (7). Moreover it was established that miR-26b might be putative target of NF-κB/MMP-9/VEGF mediating metastasis in NSCLC cells (7).

### Colorectal Cancer

Colorectal cancer (CRC) is a common cancer in which prognosis is associated with the degree of tumor invasion and metastasis. Dong et al. performed reverse qRT-PCR and immunohistochemistry for detection of MIEN1 mRNA and protein in colorectal cancer and normal/benign adjacent tissue (3). MIEN1 overexpression was associated with cancer aggressiveness and CRC metastasis more frequently in the lymph nodes (3). These results establish the role of MIEN1 expression in CRC, indicating that it and could be developed as a biomarker and/or therapeutic target.

### Oral Cancer

Oral squamous cell carcinoma (OSCC) is a malignancy of epithelial cells. Higher MIEN1 expression was reported in OSCC which correlates with cell invasion, migration and filopodia formation (6). Rajendiran et al. demonstrated overexpression of MIEN1 in dysplastic oral keratinocyte cells which results in the up-regulation of Akt/NF-κB effectors *viz*.MMP-9, uPA and VEGF involved in metastasis and invasion (6). These results suggest an important role of MIEN1 overexpression in OSCC metastasis. Furthermore, differential immunohistochemical staining pattern of MIEN1 was observed in specimens derived from OSCC patient with intense staining in OSCC vs. normal tissue possessing minimal/weak staining. Findings from this study corroborated with TCGA dataset, revealing a positive association of MIEN1 expression with intermediate/high-grade cancer, low survival rate and smoking habit (6).

### Gastric Cancer

Gastric cancer is the fifth most common disease worldwide and one of the leading causes of cancer-related deaths (4). Although incidence of gastric cancer has been on the decline over the past few years, resistance to chemotherapy and development of metastasis remains the major cause of patient mortality. Adenocarcinoma of the stomach and esophagus (upper gastrointestinal adenocarcinoma, UGC) is another important cause of cancer death. The 17q12 chromosomal region has been associated with various cancers including UGC. In a study, Maqani et al. noted amplified 17q12-q21 region in normal and UGC specimens where MIEN1 overexpression was frequently associated with invasive UGC tissue samples (55). Additional studies are required to substantiate these findings.

### Ovarian Cancer

Leung et al. reported association of MIEN1 protein with ovarian cancer. High levels of MIEN1 are involved in ΔNp73-mediated cisplatin drug resistance in ovarian cancer (5). Reports on MIEN1 and ovarian cancer thus far are limited. There is a pressing need to design in-depth studies to determine the role of MIEN1 in pathophysiology of ovarian cancer in relation to metastasis.

## MIEN1 and Other Diseases

Crohn's disease (CD) and ulcerative colitis (UC) are the two most important types of inflammatory bowel diseases (IBD). Out of total 163 loci genome wide known for IBD thus far; 110 loci are susceptibly shared by CD and UC. Studies prioritized the candidate risk gene occurring in these loci. Söderman et al. examined 13 genes within the genetic region of the shared CD and UC susceptibility locus rs2872507 and reported that reduced colonic expression of *MIEN1* with other proteins such as *ERBB2, GRB7*, and *PGAP3*, resulted in inflammation (19).

## Conclusion and Future Prospects

MIEN1 typically associates with the plasma membrane possessing ITAM motif that has the potential to strongly influence cell adhesion, migration, and invasion related to EMT. Limited information is available assessing the role of MIEN1 in various human malignancies, suggesting the demand for additional well-designed studies to evaluate its functional role in cancer. A possibility exists that MIEN1 proteins can directly or indirectly interact with soluble or cellular ligands. How closely MIEN1 function(s) is associates with pathophysiology of various human disease is unclear and remains a subject for further investigation. Higher MIEN1 expression mediates metastasis and invasion in malignant cells, indicating that the protein could be developed as a biomarker and/or therapeutic target. Although the solution structure of MIEN1 has been established, the discovery of MIEN1 inhibitors is not preferable. *In silico* drug discovery prefers crystal structure of proteins over solution structure as it provides the most stable protein conformation and high-resolution structure. This might be achieved through protein-protein interaction studies, in order to crystalize a particular protein (56). Lack of experimental evidence on MIEN1 protein-protein interaction (Figure 6) might be the reason behind lack of MIEN crystal structure to date.

Cancer drug resistance is a complex phenomenon that develops in response to chemotherapy. Studies on MIEN1 have shown its role in promoting drug resistance in some cancer types. Development of small molecule inhibitors specifically targeting MIEN1 in cancer has not yet been reported. However, as structural information becomes available, and as relevant molecular interactions are identified, this approach should become more feasible. We hereby recommend exploration and validation of natural and/or synthetic inhibitors of MIEN1 that might directly target this protein and/or its posttranslational modification. In this direction, the small size of MIEN1 protein might be one of the challenges associated with therapeutic drug targeting.

Use of antibodies or antibody-derived agents can be therapeutic target to cancer. In general, MIEN1 specific antibodies might have therapeutic potential in cancer treatment by suppressing important functions of MIEN1 or its associated proteins. It is still unknown whether MIEN1 has a role in normal cell development. Any association of MIEN1 with normal cell development will lead to challenges in acquiring tumor cell specificity. Another issue is the lack of information on cellular receptors for MIEN1 proteins. Consequently, downstream effects of MIEN1 knockdown, other than metastasis, requires further studies. As MIEN1 functional protein processing involves GGtase-1 enzyme, design of new novel inhibitor(s) of GGtase-1 may be another avenue to obstruct MIEN1 protein.

MIEN1 has been identified as a primary regulator of cancer cell migration and invasion. Emerging literature suggest that this protein is overexpressed in various human cancers including breast, prostate, colorectal, gastric, ovarian, squamous cell carcinoma and non-small cell lung cancer and in inflammatory bowel diseases. However, additional studies are needed to establish the involvement of MIEN1 in other types of cancers such as brain tumors, melanoma, hematological malignancies and other inflammatory disorders.

In conclusion, advances in the understanding of MIEN1 biochemistry and MIEN1 tumor biology over years, together with technological advancement in therapeutic targeting approaches, should lead to further *in vivo* validation of the anticancer benefits of targeting MIEN1 and associated proteins.

## Author Contributions

SK designed and conceptualized the study. SK, SG, and PK have made substantial contributions to conception and writing of the manuscript. The figures and tables were developed by SK, SG, AS, and PK.

### Conflict of Interest Statement

The authors declare that the research was conducted in the absence of any commercial or financial relationships that could be construed as a potential conflict of interest.
